# Contour Information-Guided Multi-Scale Feature Detection Method for Visible-Infrared Pedestrian Detection

**DOI:** 10.3390/e25071022

**Published:** 2023-07-04

**Authors:** Xiaoyu Xu, Weida Zhan, Depeng Zhu, Yichun Jiang, Yu Chen, Jinxin Guo

**Affiliations:** National Demonstration Center for Experimental Electrical, School of Electronic and Information Engineering, Changchun University of Science and Technology, Changchun 130022, China; cust-xxy@mails.cust.edu.cn (X.X.); zhudepeng@mails.cust.edu.cn (D.Z.); jiangyichun@mails.cust.edu.cn (Y.J.); chenyu@mails.cust.edu.cn (Y.C.); guojinxin@mail.custom.edu.cn (J.G.)

**Keywords:** pedestrian detection, deep learning, infrared images, contour guidance

## Abstract

Infrared pedestrian target detection is affected by factors such as the low resolution and contrast of infrared pedestrian images, as well as the complexity of the background and the presence of multiple targets occluding each other, resulting in indistinct target features. To address these issues, this paper proposes a method to enhance the accuracy of pedestrian target detection by employing contour information to guide multi-scale feature detection. This involves analyzing the shapes and edges of the targets in infrared images at different scales to more accurately identify and differentiate them from the background and other targets. First, we propose a preprocessing method to suppress background interference and extract color information from visible images. Second, we propose an information fusion residual block combining a U-shaped structure and residual connection to form a feature extraction network. Then, we propose an attention mechanism based on a contour information-guided approach to guide the network to extract the depth features of pedestrian targets. Finally, we use the clustering method of mIoU to generate anchor frame sizes applicable to the KAIST pedestrian dataset and propose a hybrid loss function to enhance the network’s adaptability to pedestrian targets. The extensive experimental results show that the method proposed in this paper outperforms other comparative algorithms in pedestrian detection, proving its superiority.

## 1. Introduction

Infrared pedestrian detection algorithms are a core technology for applications, such as surveillance monitoring [1], surveillance tracking [2], and autonomous driving [3], and are a prerequisite technology for tasks such as pedestrian re-identification and pedestrian retrieval. Infrared cameras use thermal imaging technology to capture infrared images. They detect infrared radiation emitted or reflected by objects. However, the resolution and contrast of infrared images are often low due to the following reasons: (1) Infrared cameras use infrared detectors to detect infrared radiation. Common infrared detectors include thermocouples, focal plane arrays, etc. (2) When infrared radiation is irradiated by an infrared detector, the detector converts the infrared radiation into electrical signals. The electrical signals are then amplified and converted into digital signals. (3) The infrared camera transmits the digital signals to a computer or monitor. Therefore, it is difficult for infrared cameras to distinguish fine image details.

Infrared images contain only luminance information and lack color information, resulting in low contrast. Although infrared images have lower resolution and contrast, they have advantages for pedestrian detection, including (1) Infrared radiation is not affected by visible light conditions such as darkness. IR cameras can detect objects at night or in smoke. (2) IR cameras can detect pedestrians based on the thermal features of the body, which is more stable and reliable than using visible features. (3) IR images are not susceptible to light and color changes, making pedestrian detection more stable. Thus, using infrared images for pedestrian detection has both advantages and challenges.

With the continuous improvement of computing device performance and the increasing maturity of deep learning technology, deep learning-based infrared pedestrian detection methods have become the mainstream solution. These methods consistently outperform Adaboost and SVM algorithms based on Haar and HOG features, achieving higher accuracy rates, faster processing speeds, and greater scalability. For visible-IR image pedestrian detection, the focus is on utilizing paired visible-IR pedestrian images to improve the utilization of color and contrast information in visible images while retaining more spatial structure and edge information of deep features to obtain better detection results. To enhance the accuracy of pedestrian detection, it is crucial to employ efficient feature extraction modules and attention mechanisms that enable the network to focus more accurately on pedestrian target features instead of relying solely on deep fused features. Despite recent advancements in visible-IR image pedestrian detection, key challenges persist in areas such as cross-domain image alignment, robustness to lighting and weather conditions, and the accurate detection of occluded or partially obscured targets. These challenges include the following: (1) In pedestrian target detection, multi-scale feature extraction can lead to partial feature information loss. For example, at a smaller scale, some important detailed information may be lost, whereas at a larger scale, some local feature information may be lost. (2) In pedestrian target detection, multiple deep convolution and downsampling operations reduce the spatial resolution of the image, resulting in some detailed information loss, especially the detailed parts of pedestrian targets, such as contours and edges, which negatively affects the detection accuracy and recall rate of pedestrian targets.

To address the above problems, we propose a contour information-guided multi-scale feature detection method for visible-IR pedestrian detection. Our method consists of four components: image preprocessing, feature extraction, a contour information-guided attention mechanism, and a decoupled head network. Together, these components enhance the accuracy of visible-IR pedestrian detection. The paired visible-IR input images are preprocessed using two modal processing methods. One method suppresses background interference and enhances the pedestrian target contour information in IR images, whereas the other extracts color components in visible images as supplementary information in IR images. The preprocessed image features are then used to generate multi-scale fusion features that highlight significant pedestrian contours. This is achieved through a feature extraction network and a contour information-guided attention mechanism. Finally, the multi-scale fused features are fed into the decoupled head network to output accurate detection results. The main contributions of this paper are as follows:

(1) An image preprocessing method consisting of a DoG filter, Top-Hat filter, and YCrCb color space is proposed that can adequately suppress background interference, enhance texture and contour information in infrared images, and extract color information in visible images to provide high-quality input images for detection networks.

(2) A feature extraction network consisting of several multi-scale feature fusion blocks is proposed. This network can effectively extract the multi-level feature information of the image and reduce the information loss in the feature extraction process. At the same time, multi-level feature reuse is realized in the feature extraction process to reduce information redundancy.

(3) A contour information-guided attention mechanism is proposed that can extract edge information and global spatial features in parallel and fuse them to enhance edge information and spatial information. This ensures that the deep feature maps retain more detailed information and have clear edges.

(4) An anchor frame size generation method for pedestrian target detection is proposed that can better adapt to the scale variation of pedestrian targets and generate anchor frames of only the necessary size to reduce computational redundancy.

## 2. Related Works

### 2.1. Deep Learning-Based Target Detection Method

Faced with complex and diverse surveillance images, the traditional pedestrian detection algorithm (the Haar wavelet transform method [4]) exhibits poor detection of obscured pedestrian targets in complex backgrounds. The gradient direction histogram feature detection method [5] has the problems of high feature dimension and large computation, and the small-edge feature Edgelet detection method [6] exhibits poor detection of complex curves in different scale features. In recent years, deep convolutional neural networks have developed rapidly in the field of image recognition, and their detection accuracy far exceeds that of traditional classifiers. They are widely used in pedestrian detection tasks. Compared to traditional pedestrian detection systems that rely on a manual feature and classifier design, deep convolutional neural networks offer stronger nonlinear mapping capabilities and the ability to learn more robust features from large datasets. In addition, deep convolutional neural networks perform end-to-end feature learning and target classification, requiring only labeled data for training.

Ross Girshick et al. [7] proposed the R-CNN model, which used a CNN for the first time in the field of target detection. It utilized the CNN to extract the feature vector of 2000 candidate regions and then performed classification and position correction using a classifier and regressor. Compared with the sliding window method of extracting manual features, the R-CNN’s heuristic search, nonlinear mapping of CNN features, and regression correction of target frames make its detection faster and more accurate. However, the R-CNN suffers from the problem of computational redundancy, which requires resizing and CNN feature computation for a large number of overlapping candidate regions.

Ross Girshick et al. [8] proposed the Fast R-CNN target detection model in 2015, which utilizes a multi-task loss to simultaneously train target classification and detection frame regression, with two inputs and two outputs. The model inputs are the image and the estimated border of the target. Then, the feature vector of each region of interest is obtained through ROI pooling. Finally, the outputs of target classification and target border regression are obtained using two fully connected layers. The Fast R-CNN model allows for the sharing of CNN features, thereby improving detection accuracy and reducing computational overhead.

Although the end-to-end training of target detection models can be achieved using a multi-task loss, the way candidate regions are generated still affects detection efficiency. In 2016, Ren et al. [9] proposed the use of a Faster R-CNN for faster detection. The Faster R-CNN uses an additional candidate region proposal network (RPN), which replaces the previously commonly used heuristic region search method to automatically generate high-quality candidate regions and makes the RPN computation process add almost no additional memory consumption by sharing parameters with the classification and border regression networks. In addition to the improvement in detection speed, the Faster R-CNN achieved the best detection accuracy at that time on the PASCAL VOC target detection dataset.

All of the above-mentioned detection methods belong to the R-CNN family of two-stage detection models, which first generate candidate regions, then extract features from these regions, and finally perform the two tasks of target classification and detection boundary regression. The two-stage detection models require repeated alternate training of the candidate region proposal network and the target classification network, making the entire training process relatively tedious and time-consuming.

To solve the disadvantages of slow detection and the large number of parameters in the two-stage detection models, Joseph Redmon et al. [10] proposed the YOLO (You Only Look Once) target detection model in 2016. Unlike the methods in the R-CNN family, YOLO adopts the regression idea to solve the target detection problem. It takes the entire image as the input to the network and directly predicts the detection boundaries of the targets in the image, as well as the confidence of those boundaries containing targets and the respective target categories. The process of the YOLO algorithm for detecting targets is divided into three main parts: the convolutional layer, the target detection layer, and the non-maximal suppression screening layer. The entire process of the model is very simple, no longer requiring a candidate region proposal process to locate potential targets but directly using regression to simultaneously determine the target location and category, which significantly speeds up detection. Additionally, each network predicts the target window using full graph information, enabling the use of contextual information, which reduces false-alarm detection rates in the background. Later, the original authors of YOLO, as well as others, successively proposed detection models such as YOLOv2 [11] and YOLOv3 [12]. YOLOv2 improves detection accuracy while ensuring detection speed by using a new underlying network Darknet-19. It also introduces the anchor mechanism from Faster R-CNN and the idea of multi-scale training. YOLOv3 adopts a better underlying network, Darknet-53. It constructs multi-scale prediction with three frames predicted at each scale and fuses features in a hierarchical connection, thereby improving the problem of inaccurate detection of small targets while maintaining real-time detection speed.

Alexey Bochkovskiy et al. [13] proposed the YOLOv4 algorithm in 2020, which utilizes a CSPDarknet53 backbone network, SPP and PAN modules to enhance the detection of targets at different scales compared to YOLOv3, and strategies such as multi-scale training, data augmentation, and Mosaic to improve the robustness of the model. The Ultralytics [14] team proposed the YOLOv5 algorithm in 2020, which utilizes a lighter EfficientNet as the backbone network compared to YOLOv4, reducing the consumption of computational resources while improving feature extraction. It also uses data enhancement methods such as AutoAugment and Mosaic to improve the robustness of the model. Adaptive and dynamic scaling schemes, as well as optimized data augmentation, are used to improve the robustness of the model. Compared to YOLOv5, YOLOv7, which was proposed by Wang et al. [15] in 2022, uses a RepConv heavy parameter convolution to speed up the network operation while maintaining model performance. The shallow features of the head part are extracted as the Aux head, and the deep features, which are the final output of the network, are used as a guide for the Lead head. Akshatha et al. [16] evaluated the performance of different backbones of the Faster R-CNN and single-shot multi-box detector (SSD) algorithms for detecting human targets in aerial thermal images.

The general models described above can extract and learn both shallow and deep feature information, as well as semantic information, about the target. This enables them to identify statistical patterns and essential target features in the data, resulting in more accurate detection results. However, despite recent advances in pedestrian detection accuracy, false and missed detections still occur due to the challenges presented by large-scale variations in pedestrian targets and the presence of similar objects in the surrounding environment.

### 2.2. Deep Learning-Based Pedestrian Detection Contour Extraction Method

Biswas et al. [17] utilized a local steering kernel (LSK) as a low-level descriptor to detect pedestrians in far-infrared images, which can effectively capture the local image geometry. The authors also introduced a new image similarity kernel, which provides a relatively short and simple training phase to build a robust pedestrian detector. Furthermore, they utilized a multichannel discrete Fourier transform, instead of a sliding window-based detection method, to facilitate very fast and efficient pedestrian localization.

Raza Shahzad et al. [18] proposed the use of template-matching pedestrian contours for detection. After detection, the Kalman filter is used for tracking. Gavrila et al. [19] proposed a global template-matching method in which pedestrian contours of different shapes are initially stored in a database as templates. Next, edge contours are extracted from the input image, and the similarity between the contours of the input image and the pedestrian contour template is measured using a correlation metric. Finally, the detection results are output based on the magnitude of similarity. Braik [20] et al. proposed a reliable and real-time method for detecting pedestrians in image scenes with highly variable appearances. To enhance the reliability of detectable content and achieve real-time detection rates, the authors utilized a combination of visual cues, edge-based features, and color information as the basis for training a cascaded random forest (RF) classifier for detecting local contour cues in pedestrian images. Shen et al. [21] proposed a method for campus pedestrian image detection using HSV thresholding binarization, image morphology processing, and image contour detection fitting. The method involves the use of erosion and extension operations, along with the adjustment of different rectangular structure elements, to reduce noise in the surroundings and extract campus pedestrian contours. Razzok et al. [22] applied multiple edge filters to locate contour cues and extract contours from various features, including the census transform (CT), modified census transform (MCT), and local gradient pattern (LGP) from the image. They accomplished this without utilizing any image recovery algorithms.

### 2.3. Improved Pedestrian Detection Method Based on Deep Learning

Song et al. [23] proposed a robust multispectral feature fusion network (MSFFN) for pedestrian detection. The network integrates two modal image features using corresponding multi-scale semantic feature extraction modules for visible and infrared pedestrian images.

Zhang et al. [24] proposed an attention-based multilayer fusion network, which includes a channel attention module (CAM) and a spatial attention module (SAM), incorporated into a three-stream deep convolutional neural network architecture. This network enables a more subtle adjustment of the weights of multispectral features in the channel and spatial dimensions, respectively.

Jet al. [25] proposed a multi-scale attention mechanism to improve the extraction of distinguishable depth features for high-overlap targets. Liu et al. [26] proposed predicting dense pedestrian density, setting non-maximal suppression (NMS) thresholds according to the denseness of different regions, and increasing low-confidence candidate frames. C et al. [27] proposed being able to better distinguish the target from the background by enhancing background feature information. Yang et al. [28] proposed parallel branching using pooling of interest with partial awareness to handle larger- or smaller-sized targets. Wang et al. [29] proposed a repulsion loss among pedestrians in a crowd scene and improved pedestrian localization accuracy by adding penalty terms to make the prediction frame as close as possible to the corresponding target’s real frame and away from other targets. Zhang et al. [30] proposed a loss function (AggLoss) and occlusion-aware RoI pooling to allow the model to learn different parts of pedestrian instances to localize the target. To address severe occlusion problems, methods for jointly training different patterns have emerged. Zhang et al. [31] proposed a visible-region generation attention mechanism to be used as external supervision for learning occlusion patterns. Zhao et al. [32], by combining elliptic Fourier descriptors and normalized central moments, proposed elliptic Fourier and moment descriptors (EFMD) to describe moving target contours. Zhang et al. [33] proposed using K-means clustering in the training set to find the best prior and improve detection accuracy. Liu et al. [34] proposed a path aggregation network consisting of a PixelShuffle-based (Shuffle-Panet) and an effective pyramidal convolutional block attention module (EPA-CBAM) to improve the detection performance of small and occluded pedestrian targets.

## 3. Proposed Algorithm

**Figure 1:**
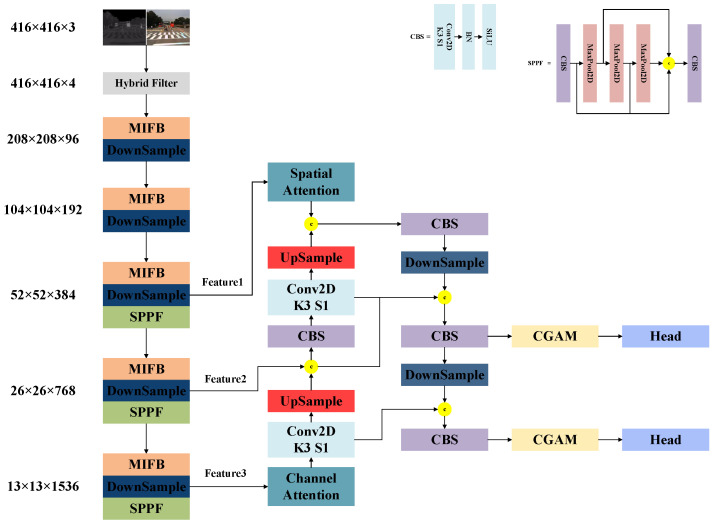
Framework of the network proposed in this paper.

A schematic diagram of our proposed network is shown in Figure 1. The network consists of four parts: image preprocessing, feature extraction, a contour information-guided attention mechanism, and a decoupled head network. Next, we describe the working principle, design idea, and specific implementation of each part.

### 3.1. Image Preprocessing

**Figure 2:**
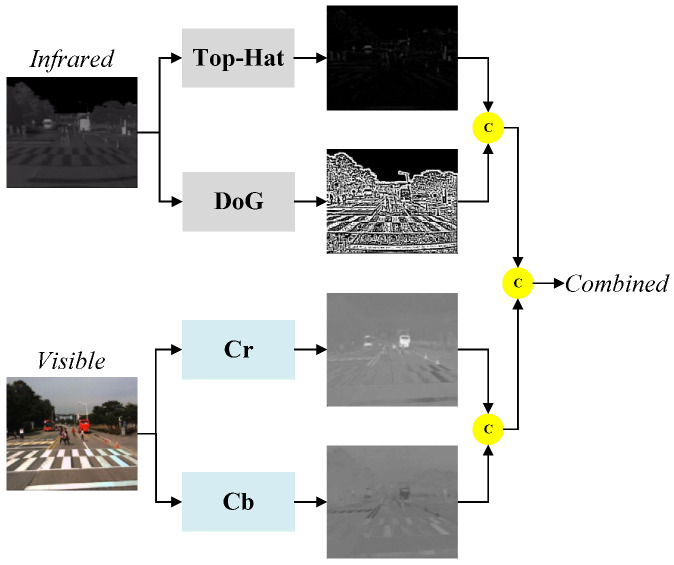
Image preprocessing method.

An infrared image is an image captured using an infrared camera that contains only one infrared radiation value per pixel, whereas a visible image contains rich color information per pixel. However, in infrared pedestrian images that contain complex backgrounds, it is difficult to detect pedestrian targets that do not have distinct contour shapes when only using the deep feature extraction network approach. To solve this problem, we propose an image preprocessing method that can be applied to IR pedestrian target detection. The method can enhance the contour information of the pedestrian target in the input IR image, suppress background interference, and extract the color components in the visible image as the complementary information of the IR image, providing a high-quality input image for the subsequent network. The image preprocessing method proposed in this paper is shown in Figure 2. It consists of a Top-Hat filter [35], Difference of Gaussians (DoG) [36] filter, and YCrCb [37] color space to enhance the contour information, suppress background interference, and display the contour and structure of the pedestrian target more clearly by using the color information from the visible image. The Top-Hat morphological filter is a nonlinear filter that can preserve the infrared pedestrian target and highlight the target when the template size is slightly larger than the target size. Through experimental comparison, the template size of the Top-Hat filter in this paper is chosen to be 19 × 19. The values of the filter parameters of the DoG filter follow a Gaussian distribution, and a smaller template size helps suppress the effect of a complex background but also weakens the energy of the IR pedestrian target. On the other hand, a larger template size enhances the ability to preserve the target energy but weakens the ability to suppress the background. To strike a balance between these two capabilities, the template size of the DoG filter used in this paper is set to 9 × 9. To make full use of the color information in the visible image, we use the YCrCb method to extract the Cb and Cr color components.

### 3.2. Feature Extraction

High-level features are mainly used to understand and infer the semantic information of target categories in images, whereas low-level features contain spatial features that retain the target contours and spatial location information. As the network continues to deepen, the features passed in the network gradually lose some spatial information due to multiple pooling operations. Therefore, when there is a lack of interaction of multi-level features within the network, it limits the ability of the network to obtain context. To solve these problems and fully exploit the multi-scale information of pedestrian targets, this paper proposes a pedestrian feature extraction network consisting of multiple connected multi-scale feature information fusion blocks, where each multi-scale feature information fusion block consists of multiple connected information fusion residual blocks.

**Figure 3:**
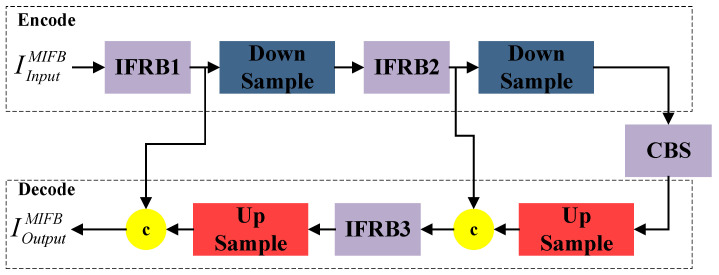
Multi-scale feature information fusion block.

As shown in Figure 3, the proposed multi-scale feature information fusion block (MIFB) consists of several information fusion residual blocks (IFRBs), upsampling layers, and downsampling layers connected in sequence. The upper part of the multi-scale feature information fusion block (MIFB) is the encoding part and the lower part is the decoding part. For the input image $I_{Input}^{MIFB}\in R^{C\times H\times W}$, the MFIB extracts the features of both scales and decodes them separately in the decoding stage to obtain the final output. The mathematical expression is as follows:

(1) $$I_{{}_{Encode}}^{MIFB}=F_{CBS}^{MIFB}\left(F_{\max}^{MIFB}\left(F_{IFRB2}^{MIFB}\left(F_{\max}^{MIFB}\left(F_{{}_{IFRB1}}^{MIFB}\left(I_{Input}^{MIFB}\right)\right)\right)\right)\right)$$

(2) $$I_{{}_{Decode}}^{MIFB}=F_{upsample}^{MIFB}\left(F_{{}_{IFRB3}}^{MIFB}\left(F_{upsample}^{MIFB}\left(I_{{}_{Encode}}^{MIFB}\right)⊕I_{{}_{IFRB2}}^{MIFB}\right)\right)$$

where $F_{{}_{IFRBi}}^{MIFB},i=1,2,3$ denotes features that are processed by the information fusion residual block; $F_{\max}^{MIFB}$ denotes the global maximum pooling operation; $F_{upsample}^{MIFB}\left(I_{{}_{Encode}}^{MIFB}\right)$ denotes the size of $I_{{}_{Encode}}^{MIFB}$, which is upsampled to the size of $I_{{}_{IFRB2}}^{MIFB}$ using bi-trivial interpolation upsampling; and $F_{upsample}^{MIFB}\left(F_{{}_{IFRB3}}^{MIFB}\left(F_{upsample}^{MIFB}\left(I_{{}_{Encode}}^{MIFB}\right)⊕I_{{}_{IFRB2}}^{MIFB}\right)\right)$ denotes the size of $F_{{}_{IFRB3}}^{MIFB}\left(F_{upsample}^{MIFB}\left(I_{{}_{Encode}}^{MIFB}\right)⊕I_{{}_{IFRB2}}^{MIFB}\right)$, which is upsampled to the size of $I_{{}_{IFRB1}}^{MIFB}$ using bi-trivial interpolation upsampling.

The pooling layer, bi-triple interpolation upsampling, and stitching operations in the channel dimension in the FIFR extract and integrate features at two scales. The MFIB is formed by stacking IFRBs, which further extract and integrate the multi-scale feature information. This progressive method of extracting and integrating the multi-scale feature information helps the network retain more spatial feature information.

**Figure 4:**
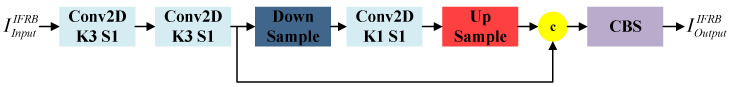
Feature information fusion residual block.

As shown in Figure 4, the information fusion residual block proposed in this paper consists of several convolutional, downsampling, and upsampling layers. For the input image $I_{Input}^{IFRB}\in R^{C\times H\times W}$, we first obtain the feature map $I_{{}_{conv1}}^{IFRB}$ using two convolutional layers, both with a kernel size of 3 × 3 and a step size of 1. Next, we use the downsampling layer to perform the scale transformation operation, and we multiply the output of the downsampling layer with a kernel size of 1 × 1 and a step size of 1 to obtain the feature map $I_{{}_{conv3}}^{IFRB}$. The small-sized feature map $I_{{}_{conv3}}^{IFRB}$ is obtained after a double-triple interpolation upsampling operation to obtain the feature map $I_{upsample}^{IFRB}$. Then, it is stitched with $I_{{}_{conv2}}^{IFRB}$ in the channel dimension and passed through the CBS module to obtain $I_{Output}^{IFRB}$. The mathematical expression for this process is as follows:

(3) $$I_{Output}^{IFRB}=F_{CBS}^{IFRB}\left(I_{{}_{conv2}}^{IFRB}⊕F_{upsample}^{IFRB}\left(I_{{}_{conv3}}^{IFRB}\right)\right)$$

where ⊕ denotes the splicing operation in the channel dimension, and $F_{upsample}^{IFRB}\left(I_{{}_{conv3}}^{IFRB}\right)$ denotes the upsampling of the size of $I_{{}_{conv3}}^{IFRB}$ to match the size of $I_{{}_{conv2}}^{IFRB}$ using the dual cubic interpolation upsampling method. Finally, the splicing results are sequentially passed through the CBS convolution block to obtain the output result $I_{Output}^{IFRB}$ of the IFRB.

**Figure 5:**
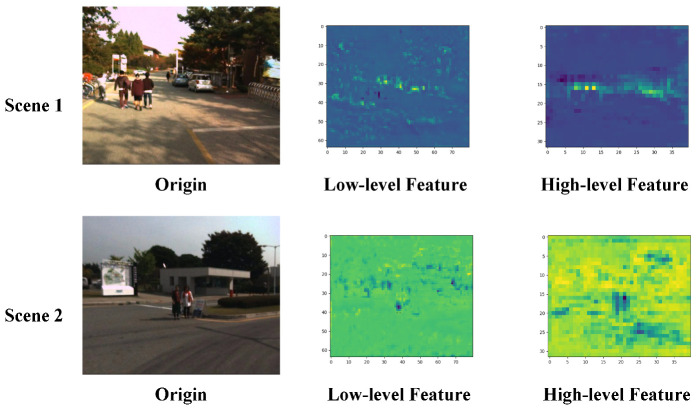
Visualization results of the feature extraction.

We randomly selected two scene input networks to observe the characteristics of different scale feature maps in the feature information fusion residual blocks and the visualization results are shown in Figure 5. The shallow features of Scene 1 and Scene 2 show pedestrians in the input images, along with rich background information. These features provide supplementary information that can be jointly used with the deep features to detect target locations. On the other hand, the deep features of Scene 1 and Scene 2 focus more on the expression of deep pedestrian semantic information. They reduce the ratio of the background to other target features and provide high-quality deep pedestrian target semantic information for subsequent detection.

### 3.3. Contouring Information to Guide the Attention Mechanism

**Figure 6:**
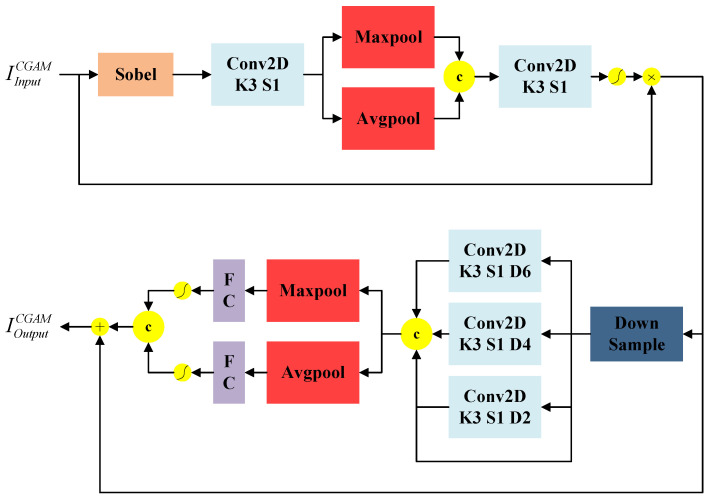
Schematic diagram of the structure of the expanded convolutional attention module guided by the target contour information.

The low-level image information contains rich contour and spatial location information. However, the deep features obtained through the multi-scale feature information fusion-block processing lose some of the spatial information and contour features of the pedestrian targets. Therefore, the traditional attention mechanism cannot accurately calculate the relationship between pixels, making the pedestrian detection network unable to predict pedestrian targets with clear boundaries. To solve these problems, this paper introduces a channel attention mechanism with null convolution to improve the traditional spatial attention mechanism by embedding attention-guided local details and global semantics to balance the local details and global semantic information. As shown in Figure 6, the contour information-guided attention mechanism (CGAM) consists of two parts. The first part is a target contour information guidance module for extracting and fusing pedestrian target contour information. The second part is a channel attention module with three dilated convolutions.

The target contour information guidance module extracts the contour information $I_{sobel}^{CGAM}$ of the pedestrian target in the input image $I_{Input}^{CGAM}\in R^{C\times H\times W}$ using a Sobel convolution. It subsequently passes through the global maximum pooling layer and performs the mean pooling operation along the channel dimension. Then, it performs the convolution operation on the generated global maximum pooling result $I_{Maxpool}^{Channel}\in R^{C\times 1\times 1}$ in the channel dimension, and the mean pooling operation result $I_{Avgpool}^{Spatial}\in R^{1\times H\times W}$ in the channel dimension after stitching. Finally, it multiplies it with the input feature $I_{Input}^{CGAM}\in R^{C\times H\times W}$ after the sigmoid operation to obtain the target contour information guidance feature $I_{{}_{Edge}}^{CGAM}\in R^{1\times H\times W}$. The mathematical expressions for the target profile information guidance module are given by

(4) $$I_{{}_{sobel}}^{CGAM}=F_{conv1}^{CGAM}\left(F_{sobel}^{CGAM}\left(I_{Input}^{CGAM}\right)\right)$$

(5) $$I_{{}_{Edge}}^{CGAM}=F_{{}_{Activate}}^{CGAM}\left(F_{conv2}^{CGAM}\left(I_{Maxpool}^{Spatial}⊕I_{Avgpool}^{Spatial}\right)\right)$$

where $F_{{}_{Activate}}^{CGAM}$ denotes the activation function, and $F_{sobel}^{CGAM}$ denotes the Sobel operator edge operation.

To reduce computational effort and save limited computational resources, the fourfold downsampling operation is performed on $I_{{}_{Edge}}^{CGAM}\in R^{1\times H\times W}$ to obtain the dilation convolution result $I_{{}_{Down}}^{CGAM}\in R^{C\times H/4\times W/4}$. Subsequently, a null convolution with a convolution kernel size of 3 × 3 and dilation rates of 2, 4, and 6 is introduced. The input and output of the null convolution are stitched in the channel dimension and then subjected to global maximum pooling and mean pooling operations in the spatial dimension to obtain $I_{Maxpool}^{Channel}\in R^{C\times H/4\times W/4}$ and $I_{Avgpool}^{Channel}\in R^{C\times H/4\times W/4}$, respectively. They are then fully concatenated and processed with an activation function to obtain the features $I_{c}^{max}$ and $I_{c}^{avg}\in R^{C/2\times 1\times 1}$, respectively. Afterward, the features $I_{c}^{max}$ and $I_{c}^{avg}\in R^{C/2\times 1\times 1}$ are processed by the FC fully connected layer and Sigmoid function, respectively, to obtain the contribution weights $weight_{max}$ and $weight_{avg}$ for different channels. Finally, the two contribution weights are combined through a weighted sum, processed with the Sigmoid function, and multiplied with the feature $I_{{}_{Edge}}^{CGAM}$ to obtain the final feature $I_{Output}^{CGAM}$.

**Figure 7:**
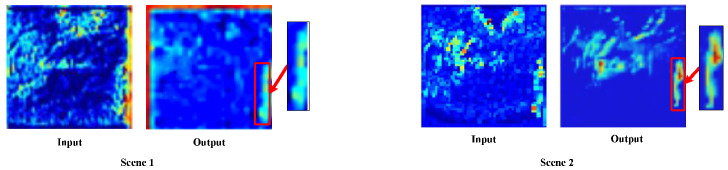
Visualization results of CGAM.

In order to analyze the actual operation mode of the CGAM, we visualized the results of the input and output feature maps of the CGAM, as depicted in Figure 7. As seen in the figure, the pedestrian target feature contours in the input feature maps of Scene 1 and Scene 2 are not obvious, and the background features contain multiple target features, resulting in an incomplete representation of the entire pedestrian target information. However, after CGAM processing, the pedestrian target contour features in the output feature maps of Scene 1 and Scene 2 are more prominent and smooth. This effectively eliminates the influence of interference and helps improve the target detection accuracy.

### 3.4. Decoupled Head Network and Loss Function

In traditional target detection algorithms, the head network part is usually composed of multiple convolutional and fully connected layers with a large number of parameters. Although the head network of the YOLO series of algorithms can solve the classification and regression problems simultaneously, the two subtasks are interdependent, which significantly affects network convergence. Additionally, there is an issue of spatial misalignment between them, which can result in the network requiring a large number of parameters and computational resources, making it prone to overfitting.

**Figure 8:**
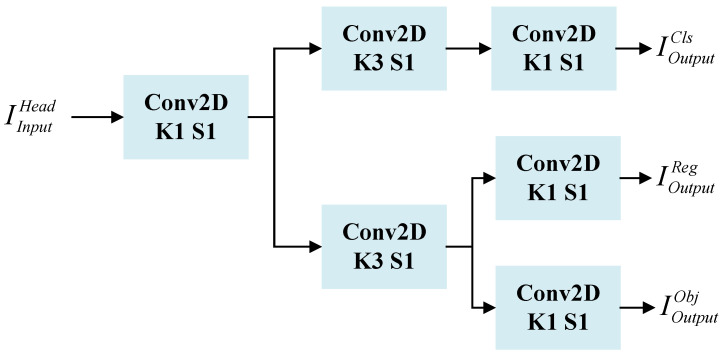
Schematic diagram of the structure of the detection head.

To solve the above problems, as shown in Figure 8, the decoupled head network structure proposed in this paper uses a decoupled head decoding network for classification and regression. It first utilizes a 1 × 1 convolution to downscale the features of the attention mechanism, which is guided by contour information. Then, it employs two 3 × 3 convolutions in each of the classification and regression branches to reduce the number of parameters. Meanwhile, the decoupling operation enables handling different detection tasks separately. This allows for better adaptation to different target scales and shapes, thereby improving the accuracy of detection.

Traditional loss functions for target detection rely on aggregated bounding box regression metrics such as the distance, overlap region, and aspect ratio between the prediction box and the true box (i.e., GIoU [38], CIoU [39], ICIoU [40], etc.). However, the methods proposed and used so far do not take into account the directional problem of the mismatch between the true and predicted frames. This deficiency leads to slower and less efficient convergence, probably due to the phenomenon of bounding box instability. During the training process, the predicted bounding boxes exhibit unstable motion, which eventually leads to the generation of suboptimal models. To solve the above problems, this paper introduces the SIoU [41] loss function as the loss function of the detection frame regression. The SIoU loss redefines the distance loss and introduces the vector angle between regressions, which effectively reduces the regression degrees of freedom, speeds up the network convergence, and further improves regression accuracy.

The SIoU loss function consists of the angle cost, distance cost, shape cost, and IoU. It can be represented as follows:

(6) SIoU=Distancecost(angle+distance)+Shapecost+IoUloss

(7) $$SIoU=1-IoU+\frac{\Delta +\Omega }{2}$$

The angle cost is defined as follows:

(8) $$L=1-2*sin^{2}\left(arcsin\left(x\right)+\frac{\pi }{4}\right)$$

where $x=\frac{c_{h}}{\sigma }=sin\left(\alpha \right)$, $sigma=\sqrt{{\left(b_{c_{x}}^{gt}-b_{c_{x}}\right)}^{2}+{\left(b_{c_{x}}^{gt}-b_{c_{y}}\right)}^{2}}$, $c_{h}=max\left(b_{c_{y}}^{gt},b_{c_{y}}\right)-min\left(b_{c_{y}}^{gt},b_{c_{y}}\right)$, sigma denotes the distance between the center points of the real pedestrian frame and the predicted pedestrian frame; $c_{h}$ denotes the height difference between the center point of the real pedestrian frame and the predicted pedestrian frame; $b_{c_{x}}^{gt},b_{c_{y}}^{gt}$ denote the center coordinates of the real pedestrian frame; and $b_{c_{x}},b_{c_{y}}$ denote the coordinates of the center of the predicted pedestrian frame.

The distance cost is defined as follows:

(9) $$\Delta =\sum_{t=x,y}\left(1-e^{-\gamma \rho_{t}}\right)$$

where $\rho_{x}={\left(\frac{b_{c_{x}}^{gt}-b_{c_{x}}}{c_{w}}\right)}^{2}$, $\rho_{y}={\left(\frac{b_{c_{y}}^{gt}-b_{c_{y}}}{c_{h}}\right)}^{2}$, gamma=2−Λ, $c_{w},c_{h}$ denote the width and height of the minimum outer rectangle of the real pedestrian frame and predicted pedestrian frame, respectively.

The shape cost is defined as follows:

(10) $$\Omega =\sum_{t=w,h}{\left(1-e^{-\omega_{t}}\right)}^{\theta }={\left(1-e^{-\omega_{w}}\right)}^{\theta }+{\left(1-e^{-\omega_{h}}\right)}^{\theta }$$

where $\omega_{w}=\frac{|w-w^{gt}|}{max(w,w^{gt})}$, $\omega_{h}=\frac{|h-h^{gt}|}{max(h,h^{gt})}$, w,h denote the width and height of the predicted pedestrian frame; $w^{gt},h^{gt}$ denote the width and height of the real pedestrian frame; and θ controls the level of attention to the shape loss.

### 3.5. Improved Anchor Frame Size

The anchor frame size used for multi-scale detection in the YOLO family of algorithms is based on the fixed cluster size generated from the COCO dataset [42]. However, the pedestrian target sizes in the KAIST pedestrian dataset are different from the COCO dataset. Since the pedestrian targets in the dataset used in this paper are of a single type and do not vary much from one target size to another, we combined the clustering and IoU metrics to design an improved anchor frame-size generation method that can be applied to the KAIST pedestrian dataset.

**Figure 9:**
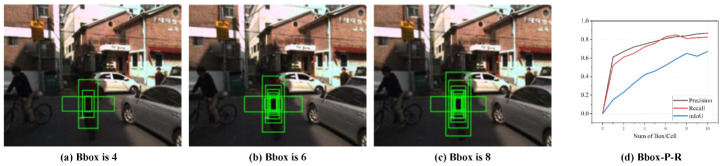
Schematic diagram of some single cells with different numbers of anchors. (**a**) Number of anchors is 4. (**b**) Number of anchors is 6. (**c**) Number of anchors is 8. (**d**) Detection accuracy (green solid line) and recall (red solid line) curves versus the number of bounding boxes per cell (from 1 to 10).

The K-means clustering algorithm [43] requires the initialization of K cluster centers before formal clustering. This clustering algorithm heavily depends on the initialization of cluster centers. Pedestrian targets in the KAIST pedestrian dataset suffer from large-scale variations, target overlap, and uneven distribution. If the Euclidean distance in the K-means clustering algorithm is used to determine the anchor box size of the KAIST pedestrian dataset, it will lead to the generation of anchor box sizes biased toward a larger number or larger size of pedestrian target boxes. The K-means++ clustering method [44] improves the initial value selection strategy based on the K-means method, which no longer only tends to the local optimal solution but tends to the global optimal solution. When determining the distance of the anchor frame, the Euclidean distance is usually used as a measure. However, the Euclidean distance is not able to accurately capture the differences between pedestrian targets of different shapes and sizes. In addition, the Euclidean distance is a method used to measure the distance between points in space and cannot consider the overlapping area between the target area and the anchor frame. If a pedestrian target has a similar size to the selected anchor frame size but is located away from the anchor frame, using the Euclidean distance as the criterion for anchor frame size selection will lead to false detection. The IoU, as a measure of the overlap between the detection frame and the real frame, can better consider the overlap area between the target area and the anchor frame. In addition, the IoU can adaptively adjust the size and position of the anchor frame, which is beneficial for accommodating targets of different shapes and sizes. We combined the advantages of the above-mentioned methods by choosing the K-means++ clustering algorithm and using the average intersection ratio as the evaluation criterion for generating the anchor frame size, instead of the Euclidean distance. A higher average intersection ratio indicates a better clustering result. As shown in Figure 9a–c, we randomly selected a pedestrian target of an image in the KAIST pedestrian dataset to demonstrate the anchor box size when the number of anchor boxes was 4, 6, and 8. The calculation of the average intersection of the concatenation and distance measures is as follows:

(11) $$IoU=\frac{B\cap B^{gt}}{B\cup B^{gt}}$$

(12) $$AIoU\left(b,c\right)=\frac{1}{1+K+N_{i}}\sum_{i=1}^{K}\sum_{j=1}^{N_{i}}IoU(b,c)$$

(13) D(b,c)=1−AIoU(b,c)

where $B^{gt}$ denotes the actual area of the object’s bounding box, *B* denotes the detection area of the object’s bounding box, *b* denotes the bounding box, *c* denotes the clustered mass centers, *K* denotes the number of mass centers, $N_{i}$ denotes the number of samples for each mass center, and IoU(b,c) denotes the intersection over union between the bounding box and the mass center of the cluster.

In addition, different numbers of predicted bounding boxes in each cell have different degrees of impact on the algorithm’s performance. For the test set of the KAIST pedestrian dataset, we conducted comparison experiments to verify the impact of the algorithm’s performance, with the number of bounding boxes ranging from 1 to 10. As shown in Figure 9d, the precision, recall, and mIoU values gradually increased with the number of bounding boxes in each cell. However, the recall value reached its highest value when the number of bounding boxes was equal to seven and then gradually decreased. When the number of bounding boxes in each cell was equal to eight, the increases in the precision and recall values tended to slow down, and when the number of bounding boxes in each cell was equal to nine, the precision and recall values tended to be stable and unchanged. When the number of bounding boxes in each cell was less than or equal to eight, the mIoU value showed an increasing trend. When the number of bounding boxes was equal to nine, the mIoU value suddenly decreased. When the number of bounding boxes was equal to 10, the mIoU value increased slightly.

In summary, to balance the precision, recall, and mIoU, we set eight bounding boxes in each cell. The sizes of the eight bounding boxes were (23,50); (29,66); (37,82); (44,106); (58,137); (64,43); (81,203); and (209,56).

## 4. Experiments

In this section, we describe a series of experiments that were conducted to evaluate the effectiveness of our proposed method. First, we describe the dataset and the evaluation metrics used. Second, in Section 4.2, we conduct a qualitative and quantitative evaluation of the performance of the proposed method on the KAIST [45] pedestrian dataset. Third, in Section 4.3, we conduct a qualitative and quantitative evaluation of the performance of the proposed method on the OSU [46] Thermal-Color dataset. Finally, in Section 4.4, we present the results of ablation experiments for the image preprocessing method, feature extraction, contour-guided attention mechanism, and improved anchor frame size.

### 4.1. Dataset and Experimental Data Setup

#### 4.1.1. Dataset Settings

**Figure 10:**
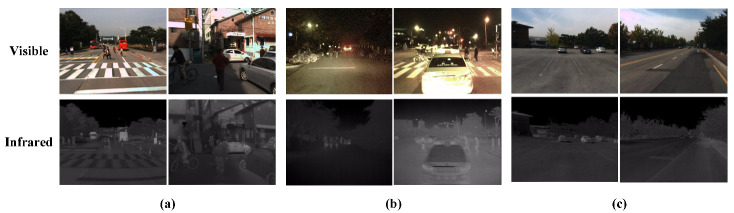
Selected images of the KAIST pedestrian dataset. (**a**) Effectively aligned visible-IR image pairs with pedestrian targets in daytime conditions; (**b**) effectively aligned visible-IR image pairs with pedestrian targets in nighttime conditions; (**c**) aligned visible-IR image pairs with no pedestrian targets in daytime conditions.

All experimental datasets used were from the KAIST multispectral pedestrian dataset. The dataset contains a total of 95,328 visible-IR image pairs of which 50,172 pairs were used for training and 45,156 pairs were used for validation. The images were obtained using a high-definition visible light camera and a long-wave infrared camera. We traversed all labeled files in the dataset and excluded poorly labeled and untargeted image pairs. In addition, we sampled every two frames from the training video according to the annotation sampling procedure to exclude image pairs with heavily obscured pedestrian targets and small sizes. Through the filtering and cleaning operations, we ensured that there were enough positive samples in the final training and validation datasets. The training dataset contained 7601 images, and the test dataset consisted of 2252 image pairs obtained by sampling once every 20 frames. We randomly selected four sets of visible-IR image pairs from the filtered and cleaned dataset, which included both daytime and nighttime cases, with at least three pedestrian targets in each set to maintain a balance of positive and negative samples in the dataset (see Figure 10a,b for details). We also randomly selected two sets of visible-IR images from the dataset before filtering and cleaning, both of which had no pedestrian targets, as shown in Figure 10c.

The OSU Thermal-Color dataset contains pairs of visible and infrared images of pedestrians at two locations and in seven different scenes. We selected 200 discrete frames based on different locations, scenes, and shooting times to verify the generalizability of the algorithm in this paper.

#### 4.1.2. Evaluation Indicators

For a comparison of our proposed method with state-of-the-art techniques, we used standard target detection evaluation metrics [23], including Accuracy, Recall, Precision, mAP, F1 [16], true positive (TP), false positive (FP), and false negative (FN).

We evaluated the detection results by calculating the intersection over union (IoU) of the ”predicted boundaries” and ”true boundaries”, which is the ratio of their intersection to their union. We employed different IoU thresholds: if the IoU between the predicted boundary box and the true boundary box was greater than the threshold, we classified the predicted bounding box as positive; otherwise, it was classified as negative.

TP refers to the number of correctly predicted foreground pixels, i.e., pedestrian target areas; FP refers to the number of incorrectly predicted background pixels, i.e., background areas mistaken for pedestrian target areas. For each image, the number of FPS was in the range of $\left[10^{-2},100\right]$. FN refers to the number of foreground pixels incorrectly identified as background pixels.

Accuracy refers to the percentage of all forecasts that were correctly predicted. The formula for the Accuracy rate is as follows:

(14) $$Accuracy=\frac{TP+TN}{N}$$

where *N* denotes the total number of predicted samples.

Recall refers to the ability of a model to correctly detect all real existing targets and is related to the ratio between the number of correctly detected targets and the number of all real existing targets. The formula for the Recall rate is as follows:

(15) $$Recall=\frac{TP}{TP+FN}$$

Precision refers to the proportion of true examples predicted by the model among all the predicted positive examples. In short, it measures the Accuracy of the model in identifying positive cases. Specifically, assuming that the model predicts a set of test samples consisting of both true cases (ground truth) and false-positive cases, Precision is calculated as:

(16) $$Precision=\frac{TP}{TP+FP}$$

AP represents the average value of the precision of the model at each recall level. The area under the curve formed by the precision and recall curves reflects the AP, where a larger area indicates a higher AP and better detection performance.

mAP calculates the average of the AP values across different categories and serves as a performance metric for multi-classifiers.

The F1 is one of the most commonly used metrics for evaluating model performance, particularly in binary classification tasks, where detected targets are considered positive samples and undetected targets are considered negative samples. The F1 can then be used to evaluate the performance of the model on positive and negative sample classifications. The F1 takes into account the Accuracy and Recall of the model and is calculated as:

(17) $$F1=\frac{2\times (Precision\times Recall)}{Precision+Recall}$$

#### 4.1.3. Experimental Environment

We trained our proposed network in the following hardware environment: Intel(R) Core(TM) i9-10850K with 32.0 GB RAM and an NVIDIA GeForce RTX 3090 GPU. The software platform was Windows 11 OS with Pycharm 2022.3.2, and the deep learning framework used was Pytorch 1.10.1. We trained each detector using the same procedure and hyperparameters using stochastic gradient descent: adjusting the input image size to 416 × 416, setting the batch size to 8, using the Adam optimizer, and setting the learning rate to 1 × $10^{-4}$. During the fine-tuning period, we reduced the learning rate by a factor of 10 every 5 periods and stopped training after 10 periods.

### 4.2. KAIST Pedestrian Dataset Results

**Figure 11:**
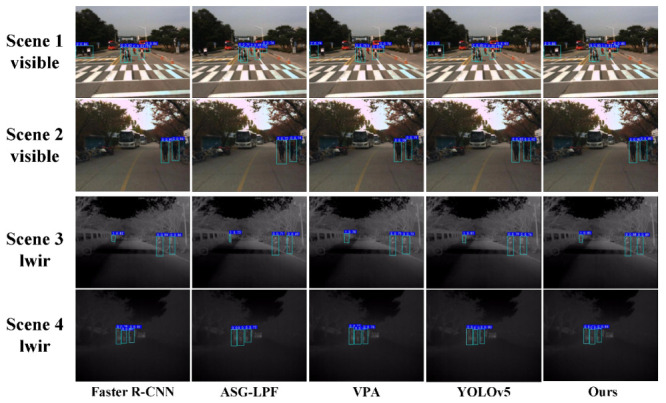
Detection results of multiple algorithms on KAIST pedestrian dataset.

Figure 11 shows the results of the qualitative analysis of our algorithm, as well as other commonly used algorithms, on the KAIST pedestrian dataset. We simplified the target categories of the KAIST pedestrian dataset, and ”pedestrian” and ”crowd” were simplified to ”0” and ”2”, respectively. We observed that our algorithm achieved better detection results in the daytime and nighttime cases, with no false or missed detections, and the prediction confidence of all pedestrian targets was better than the other methods. The Faster R-CNN and YOLOv5 algorithms achieved higher detection confidence, but there was a small deviation in the predicted frame from the true target for individual pedestrian targets. The results of the quantitative analysis of our proposed method and other commonly used pedestrian detection algorithms are shown in Table 1. The algorithm in this paper achieved mAP, $AP_{50}$, and $AP_{75}$ values of 89.1%, 89.4%, and 88.6%. Compared to the ASG-LPF, VPA, and YOLOv5 algorithms, the algorithm in this paper achieved an mAP value that was, respectively, 23.7%, 11.8%, and 6.6% higher; an $AP_{50}$ value that was, respectively, 23.0%, 14.3%, and 4.7% higher; and an $AP_{75}$ value that was, respectively, 20.7%, 12.6%, and 5.0% higher. Moreover, YOLOv5 was only 2 FPS faster than the proposed method. Although the Faster R-CNN method achieved the highest accuracy in terms of the mAP, $AP_{50}$, and $AP_{75}$ among all the methods, the detection speed of this paper’s method was significantly better, and the mAP, $AP_{50}$, and $AP_{75}$ values were at almost the same levels. Our method achieved the highest F1 among all the methods. In summary, the algorithm in this paper could balance detection accuracy and speed on the KAIST pedestrian dataset, and both metrics achieved the highest values among all the evaluated methods.

### 4.3. OSU Pedestrian Dataset Results

**Figure 12:**
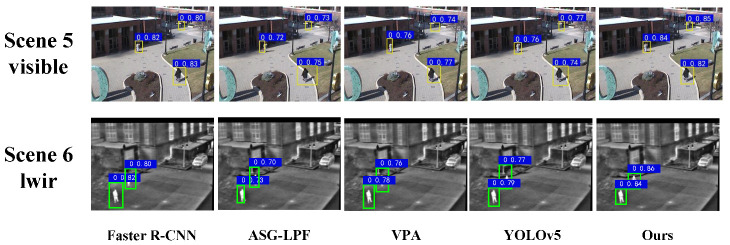
Detection results of multiple algorithms on OSU Thermal-Color dataset.

To demonstrate the generalizability of our algorithm, we compared the detection performance of our algorithm with that of other commonly used pedestrian detection methods on the OSU Thermal-Color dataset. In the images in this dataset, the camera was further away from the pedestrian targets and the image sizes were smaller, making it more difficult for the network model to detect the pedestrian targets. Figure 12 shows the results of the qualitative analysis of our algorithm and other commonly used algorithms on the OSU pedestrian dataset. As shown in Table 2, the algorithm in this paper assisted the network in highlighting the contour information of small-sized pedestrian targets through the contour information-guided attention mechanism, retaining more features of small-sized targets, thereby demonstrating a stronger detection ability for small-sized pedestrian targets. The detection speed (FPS value) of the Faster R-CNN algorithm was only 1/16 of that of the proposed method, so it could not achieve a perfect balance between detection performance and detection speed. Compared to the ASG-LPF, VPA, and YOLOv5 algorithms, our method achieved an mAP value that was, respectively, 26.2%, 14.1%, and 7.6% higher; an $AP_{50}$ value that was, respectively, 25.7%, 13.8%, and 5.2% higher; and an $AP_{75}$ value that was, respectively, 21.6%, 12.4%, and 2.6% higher. Meanwhile, in terms of detection speed, the algorithm in this paper outperformed the ASG-LPF algorithm by 16 FPS and the VPA algorithm by 7 FPS but it was almost the same as the YOLOv5 algorithm. Our method achieved the highest F1 among all the methods. In summary, the algorithm in this paper achieved the best values in the mAP, $AP_{50}$, $AP_{75}$, and FPS metrics.

**Figure 13:**
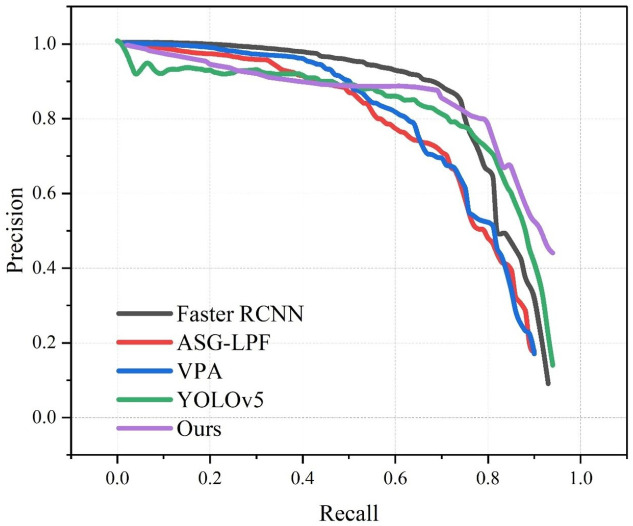
P-R of multiple algorithms on OSU Thermal-Color dataset.

In order to further demonstrate the superiority of the algorithm in this paper, the corresponding recall and precision curves of this algorithm and the commonly used algorithms are shown. As shown in Figure 13 and Table 3, in the recall and precision curves, the area under each curve represents the detection accuracy of the method, and the larger the area under the curve, the stronger the detection performance. Our method achieved the highest AUC value of the P–R curve among all the methods.

### 4.4. Ablation Experiments

To verify the effectiveness of each innovative design in the algorithm of this paper, we performed ablation experiments on the KAIST pedestrian dataset for each innovative design. We ensured that each set of experimental parameters was set identically and used a uniform 416 × 416 image size as input. By calculating the mean average precision (MAP) and recall, we evaluated the validity of each design.

We first defined a layer of ordinary convolution as $conv_{EXT}$ and $conv_{CGAM}$, both utilizing a kernel size of 3 × 3, a stride of 1, and a padding of 1. We replaced the multi-scale feature information fusion block and the contour-guided attention mechanism in the network of this paper with A and B, and we named this the baseline method. We conducted ablation control experiments by adding or substituting different modules to verify the effectiveness of all the methods proposed in this paper.

#### 4.4.1. Feature Extraction Network

To verify the effectiveness of the multi-scale feature information fusion block structure in the feature extraction network, we used a multi-scale feature information fusion block that incorporates an information fusion residual block to replace $conv_{EXT}$ in the baseline method and named it Method A.

To verify the effectiveness of the information fusion residual block in the multi-scale feature information fusion block, we replaced the information fusion residual block in the multiscale information fusion block in Method A with $conv_{EXT}$, named Method B. As shown in Table 4, compared with the baseline method, Method A has 4.0% higher detection accuracy and 4.2% higher recall in the daytime scenario and 5.0% higher detection accuracy, and 4.3% higher recall in the nighttime scenario. The reason for the improved detection accuracy and recall in both daytime and nighttime scenarios is that we use a multiscale feature information fusion block that contains information fusion residual blocks, and the increasing number of residual connections helps the feature extraction network learn features at different scales and different abstraction levels at multiple levels and enables the gradients to be passed more smoothly to the shallower levels, thus better learning of deeper features. Also, image features at different scales are fused using U-net-like connection structures, pooling layers, dual triple interpolation upsampling, and stitching operations on channel dimensions. As shown in Table 4, compared with method A, method B has a 2.1% decrease in detection accuracy, and 1.7% decrease in recall in the daytime scenario, a 2.4% decrease in detection accuracy, and a 2.1% decrease in recall in the nighttime scenario. The reason for the decrease in detection accuracy and recall in both the daytime and nighttime scenarios is that we removed the information fusion residual block from the multi-scale feature information fusion block and used $conv_{EXT}$ as a replacement. In summary, the structure of the information fusion residual block and multi-scale feature information fusion block proposed in this paper results in the gradual extraction and integration of multi-scale feature information in a manner that helps the network retain more spatial feature information.

#### 4.4.2. Contour-Guided Attention Mechanism

To verify the effectiveness of the contour-guided attention mechanism, we replaced $conv_{CGAM}$ in Method A with the contour-guided attention mechanism, which we named Method C, and replaced $conv_{CGAM}$ in Method A with the CBAM module, which we named Method D. As shown in Table 5, the addition of the CBAM in Method D resulted in a 2.3% higher detection accuracy and a 2.5% higher recall rate in the daytime scenario, as well as a 3.1% higher detection accuracy and a 2.5% higher recall rate in the nighttime scenario compared to Method A. The reason for the improved detection accuracy and recall in both the daytime and nighttime scenarios is that the CBAM attention mechanism effectively learned the spatial and channel information correlations of the input feature map and applied this information to the different levels of the feature map, effectively improving the representation capability of the network. As shown in Table 5, after the addition of the contour-guided attention mechanism, Method C achieved a 5.4% higher detection accuracy and a 7.4% higher recall rate in the daytime scenario, as well as a 6.8% higher detection accuracy and a 6.5% higher recall rate in the nighttime scenario compared to Method A. The reason for the significant improvements in the detection accuracy and recall rate in the daytime and nighttime scenarios is that we introduced a channel attention mechanism and Sobel edge extractor to the traditional spatial attention mechanism. The introduction of cavity convolution can help the network process deeper features at larger scales, improve the perceptual range of the network, and increase the feature extraction capability without increasing the number of parameters. In summary, the contour-guided attention mechanism proposed in this paper can effectively address the loss of detailed spatial structure information and edge information caused by increasing the depth of the feature layers. In addition, it can improve the attention of the convolutional neural network toward pedestrian target contours and enhance the detection capability of pedestrian targets.

#### 4.4.3. Improved Anchor Frame Size

To verify the effectiveness of the improved anchor frame size, we used the improved anchor frame size proposed in this paper and named it Method E, which was based on Method C. We used the anchor frame size of the YOLO series algorithm and named it Method F, which was also based on Method C.

As shown in Table 6, Method E achieved a 0.8% higher detection accuracy and a 1.4% higher recall rate in the daytime scenario, as well as a 1.5% higher detection accuracy and 1.9% higher recall rate in the nighttime scenario compared to Method F. The reason for the improved detection accuracy and recall rate in both the daytime and nighttime scenarios is that we combined the K-means++ and mIoU methods to generate anchor frames that were better suited for the pedestrian target size in the KAIST pedestrian dataset. This approach effectively improved the localization accuracy and recall rate for pedestrian targets, enabling the network to more accurately locate pedestrian targets and thereby improving overall detection accuracy.

#### 4.4.4. Image Preprocessing

To verify the effectiveness of image preprocessing, we added image preprocessing to Method E and named it Method G. As shown in Table 7, Method G achieved a 1.8% higher detection accuracy and a 1.3% higher recall rate in the daytime scenario, as well as a 1.4% higher detection accuracy and a 1.4% higher recall rate in the nighttime scenario compared to Method E. The improvements in the detection accuracy and recall rate in both the daytime and nighttime scenarios can be attributed to our use of image preprocessing methods to enhance contour information, suppress background interference, and extract color components from visible images for the input to the network, providing the network with high-quality input images.

To obtain the optimal template sizes for the DoG and Top-Hat filters, comparison experiments were carried out using different template sizes. Table 8 and Table 9 present the results, where we selected the average detection accuracy and recall for different template sizes. Based on these findings, we found that template sizes of 9 × 9 and 19 × 19 were the optimal template sizes for the DoG and Top-Hat filters, respectively.

## 5. Conclusions

In this paper, we propose a contour information-guided multiscale feature detection method for visible-IR pedestrian detection. The method includes an image preprocessing method, a feature extraction network, a contour information-guided attention mechanism, a decoupled head network with a loss function, and an improved anchor frame size. The image preprocessing method uses the DoG filter, Top-Hat filter, and color component extraction method YCrCb to provide high-quality input images for the network. The feature extraction network utilizes a simple U-shaped structure with a residual connection and repeated stacking to enhance the network’s ability to extract and integrate features at multiple scales. The contour information-guided attention mechanism directs the network to prioritize feature information within the pedestrian target contour region by employing a spatial-channel attention mechanism with null convolution. This mechanism embeds attention-guided local details and global semantics to balance local details and global semantic information. Furthermore, it effectively supplements the detailed information lost during convolution operations. Decoupling the head network from the loss function improves detection accuracy by calculating the directional match between the detection frame and ground truth. It also introduces the SIoU to better adapt to different pedestrian target scales and shapes. The improved anchor frame size enhances the network’s ability to adapt to pedestrian targets of varying sizes. Experiments have demonstrated that the improved anchor frame size can improve the detection accuracy of the network. Based on a comparison with various current pedestrian algorithms, our method demonstrates advantages in visual quality and quantitative criteria. It significantly improves the detection accuracy of pedestrian targets, thereby validating the application potential of the network.

## Figures and Tables

**Table 1:** Performance comparison of different advanced methods on KAIST pedestrian dataset.

Method	Backbone	FPS	F1 (%)	mAP (%)	${\mathrm{AP}}_{50}$ (%)	${\mathrm{AP}}_{75}$ (%)
Faster R-CNN [9]	VGG-16	2.8	76.26	84.6	87.6	88.1
ASG-LPF [47]	VGG-16	34	69.37	65.4	66.4	67.9
VPA [48]	CSP-Darknet-53	45	72.14	77.3	75.1	76.0
YOLOv5 [14]	New-CSP-Darknet-53	**52**	74.58	82.5	84.7	83.6
Ours	MIFB	50	**78.92**	**89.1**	**89.4**	**88.6**

**Table 2:** Performance comparison of different methods on the OSU Thermal-Color dataset.

Method	Backbone	FPS	F1 (%)	mAP (%)	${\mathrm{AP}}_{50}$ (%)	${\mathrm{AP}}_{75}$ (%)
Faster R-CNN [9]	VGG-16	3.2	74.3	86.5	87.8	85.3
ASG-LPF [47]	VGG-16	32	61.7	62.4	64.8	66.9
VPA [48]	CSP-Darknet-53	41	73.2	74.5	76.7	76.1
YOLOv5 [14]	New-CSP-Darknet-53	**50**	74.0	81.0	85.3	85.9
Ours	MIFB	48	**79.2**	**88.6**	**90.5**	**88.5**

**Table 3:** AUC of P-R.

Model	Faster R-CNN	ASG-LPF	VPA	YOLOv5	Ours
**AUC**	0.867	0.672	0.724	0.829	0.871

**Table 4:** Ablation experiments of information fusion residual blocks (IFRB) and multi-scale feature information.

Method	MAP (Day)	Recall (Day)	MAP (Night)	Recall (Night)
Baseline	73.2	71.4	70.5	71.8
Method A	77.2	75.6	75.5	76.1
Method B	75.1	73.9	73.1	74.2

**Table 5:** Ablation experiment of contour-guided attention mechanism.

Method	MAP (Day)	Recall (Day)	MAP (Night)	Recall (Night)
Method A	77.2	75.6	75.5	75.1
Method C	82.6	83.0	82.3	81.6
Method D	79.5	78.1	78.6	77.6

**Table 6:** Ablation experiments with improved anchor frame size.

Method	MAP (Day)	Recall (Day)	MAP (Night)	Recall (Night)
Method E	83.8	84.9	84.2	83.7
Method F	82.6	83.5	82.7	81.8

**Table 7:** Ablation experiments of image preprocessing methods.

Method	MAP (Day)	Recall (Day)	MAP (Night)	Recall (Night)
Method E	83.8	84.9	84.2	83.7
Method G	85.6	86.2	85.6	85.1

**Table 8:** Comparison experiments using different template sizes for the DoG filter.

Template Size	5 × 5	7 × 7	9 × 9	11 × 11
MAP	79.2	82.5	85.3	81.6
Recall	77.6	80.6	82.6	80.1

**Table 9:** Comparison experiments using different template sizes for the Top-Hat filter.

Template Size	9 × 9	13 × 13	19 × 19	21 × 21
MAP	78.4	81.4	82.3	80.2
Recall	76.5	79.6	81.0	78.6

## Data Availability

The data presented in this study are available on request from thecorresponding author.
